# Complex interactions between p.His558Arg and linked variants in the sodium voltage-gated channel alpha subunit 5 (Na*_V_*1.5)

**DOI:** 10.7717/peerj.13913

**Published:** 2022-08-17

**Authors:** Monica Lopes-Marques, Raquel Silva, Catarina Serrano, Verónica Gomes, Ana Cardoso, Maria João Prata, Antonio Amorim, Luisa Azevedo

**Affiliations:** 1 IPATIMUP-Institute of Molecular Pathology and Immunology, University of Porto, Porto, Portugal; 2 Faculty of Sciences, University of Porto, Porto, Portugal; 3 Population Genetics and Evolution, Institute of Innovation and Investigation in Health (i3S), Porto, Portugal; 4 Center for Interdisciplinary Research in Health (CIIS), Universidade Católica Portuguesa, Faculdade de Medicina Dentária, Viseu, Portugal

**Keywords:** Sodium voltage-gated channel alpha subunit 5 (Na*_V_*1.5), SCN5A variants, Genetic background, Linkage disequilibrium, Genetic modifier, Epistasis, Brugada syndrome, Cardiac channel, Cardiac diseases

## Abstract

Common genetic polymorphisms may modify the phenotypic outcome when co-occurring with a disease-causing variant, and therefore understanding their modulating role in health and disease is of great importance. The polymorphic p.His558Arg variant of the sodium voltage-gated channel alpha subunit 5 (Na*_V_*1.5) encoded by the *SCN5A* gene is a case in point, as several studies have shown it can modify the clinical phenotype in a number of cardiac diseases. To evaluate the genetic backgrounds associated with this modulating effect, we reanalysed previous electrophysiological findings regarding the p.His558Arg variant and further assessed its patterns of genetic diversity in human populations. The Na*_V_*1.5 p.His558Arg variant was found to be in linkage disequilibrium with six other polymorphic variants that previously were also associated with cardiac traits in GWAS analyses. On account of this, incongruent reports that Arg558 allele can compensate, aggravate or have no effect on Na*_V_*1.5, likely might have arose due to a role of p.His558Arg depending on the additional linked variants. Altogether, these results indicate a major influence of the epistatic interactions between *SCN5A* variants, revealing also that phenotypic severity may depend on the polymorphic background associated to each individual genome.

## Introduction

Mendelian monogenic diseases often show clinical heterogeneity, so that individuals carrying the same variant can manifest different phenotypes. This may result from epistatic interactions between variants highlighting the importance of the genetic background in the phenotypic characteristics each individual manifests (Fournier & Schacherer, 2017; Lehner, 2011; Lopes-Marques et al., 2021; Suriano et al., 2007). The importance of genetic architecture is well illustrated in the particular case of co-occurrence of polymorphic and deleterious variants in the same protein. Interactions between disease-associated variants and common polymorphisms have been reported in several proteins, including for instance the α1-syntrophin p.Ala257Gly and p.Pro74Leu variants (Cheng et al., 2011), the potassium channel KCNH2 p.Ala490Thr and p.Lys897Thr variants (Zhang et al., 2008), the glucocorticoid receptor-alpha p.Gly679Ser and p.ER22/23EK variants (Raef et al., 2008) or the DNA polymerase gamma p.Trp748Ser and p.Glu1143Gly variants (Chan, Longley & Copeland, 2006) among other examples brought together by Cooper et al. (2013). One of the most well-known case is the p.His558Arg substitution (rs1805124) in the *SCN5A* gene (Marangoni et al., 2011; Núñez et al., 2013; Poelzing et al., 2006; Viswanathan, Benson & Balser, 2003). The *SCN5A* gene is located in chromosome 3p21 and encodes the α-subunit of the primary cardiac Na^+^ channel (Na_*V*_1.5) which plays an essential role in excitation and contraction of the heart by inducing the rapid depolarization necessary to initiate action potentials (DeMarco & Clancy, 2016). There are 10 mammalian genes known to encode functionally distinct sodium channels (DeMarco & Clancy, 2016; Jiang et al., 2020). These channels are dimers of α-subunits that interact physically leading to coupled gating, in which the 14-3-3 protein is also involved (Clatot et al., 2017). Each α-subunit of the cardiac Na_*V*_1.5 is structurally organized into four domains (DI–DIV), connected by cytoplasmic linker segments (L1–L3) (Balser, 1999; Jiang et al., 2020). Each domain combines six transmembrane segments (S1–S6), where S1–S4 form the voltage-sensing module (VS), and S5–S6 and a P-loop linking them form the pore module (PM) (de Lera Ruiz & Kraus, 2015; Jiang et al., 2020).

To this date, more than 700 pathological variations have been associated to *SCN5A*, most of them are non-synonymous variants (Li et al., 2018; Verkerk, Amin & Remme, 2018). Disease-associated variants have been found in patients presenting several cardiac channelopathies (Li et al., 2018), as the Brugada syndrome (Chen et al., 2020; Glazer et al., 2020; Monasky et al., 2020; Raharjo et al., 2018), long QT syndrome type 3 (Surber et al., 2008), and other cardiac diseases such progressive cardiac conduction defect and sick sinus syndrome (SSS), atrial fibrillation, dilated cardiomyopathy, inherited arrhythmogenic cardiomyopathy (reviewed in (Rivaud, Delmar & Remme, 2020)). The p.His558Arg is a common variant in humans that was already established as a modifier of the SCN5A-associated phenotypes namely the Brugada syndrome (Marangoni et al., 2011; Núñez et al., 2013; Poelzing et al., 2006; Viswanathan, Benson & Balser, 2003), familial sick sinus syndrome (SSS) (Gui et al., 2010) and Long QT syndrome (Shinlapawittayatorn et al., 2011; Vatta et al., 2021). A number of studies showed that the effect of p.His558Arg when combined with distinct deleterious variants might be either compensating or aggravating (*e.g*. (Cheng et al., 2010; Gui et al., 2010; Marangoni et al., 2011; Núñez et al., 2013; Veltmann et al., 2016)).

Apart from p.His558Arg, likely other variants may also have modulating effects on the clinical outcome, although no studies have yet investigated the possibility (Balla et al., 2021).

Given, on the one hand, the discrepancies in the effect of p.His558Arg in the context of cardiac dysfunction and, on the other, the lack of knowledge on the possible role played by additional variants, we sought to collect and reanalyse data focusing on the p.His558Arg variant with the aim of identifying the mechanism by which intragenic molecular interactions result in distinct outcomes in electrophysiological studies.

The identification of putative additional variants with functional impact was assessed by analyses of patterns of linkage disequilibrium (LD) as well as, by dissecting the evolutionary history of the position underlying p.His558Arg in mammals, recruiting pertinent data including the recently provided by ancient DNA from archaic humans.

## Materials and Methods

### Frequency of the p.His558Arg in human populations

A literature search was conducted in PubMed database using the following keywords as queries: “H558R+SCN5A”; “H558R” and “SCN5A+modifier”. The resulting hits were manually screened, to eliminate duplicate works and to select research articles with functional assays of Na_*V*_1.5 with and without the analysis of the p.His558Arg variant (rs1805124). Details such as the genetic background in which *SCN5A* was expressed, if p.His558Arg was expressed in the same construct as the target deleterious variant (cis) or co-expressed in an independent construct (trans) and overall outcome of the functional assays, were collected (Table S1).

The rs1805124 frequency in human populations was obtained from the Ensembl release 100 variation pipeline (Hunt et al., 2018; Zerbino et al., 2017) using data from the 1,000 genome project (1KGP) phase three on the GRCh38 (Auton et al., 2015). The mean frequencies of each super population were tested performing a Bayesian one sample test of population mean allele frequencies in JASP V0.12.2 (https://jasp-stats.org/) (Morey et al., 2011; Rouder et al., 2009).

### Patterns of LD targeting the rs1805124 variant

LD analysis was conducted in the LDlink database (Machiela & Chanock, 2015) using the rs1805124 as the target SNP. An initial screen using LDtrait was conducted to obtain a list of variants associated with cardiovascular disease traits in LD with rs1805124. The window size was set as 500,000 bp and R^2^ threshold value was set to 0.1, to exclude rare variants from the analysis. Pairwise allele correlation with rs1805124 for each variant was determined using LDpair in each super population. Combined haplotypes and corresponding frequencies for the selected set of variants was obtained using the LDhap tool (Machiela & Chanock, 2015).

### Comparative analysis in archaic humans and in mammals

rs1805124 and remaining polymorphisms identified in the linkage disequilibrium analyses were next investigated in archaic human genomes: Altai Neanderthal (Prüfer et al., 2014), Vindija Neanderthal (Green et al., 2010), Siberian Denisovans (Meyer et al., 2012), Mal’ta1 (Raghavan et al., 2014), Kostenki14, Paleo Eskimo (Rasmussen et al., 2010) and Tyrolean Iceman Otzi (Keller et al., 2012). For the Altai Neanderthal, Vindija Neanderthal and Siberian Denisova data were collected in the Max Planck Institute for Evolutionary Anthropology ancient genome browser (https://bioinf.eva.mpg.de/jbrowse/) and in the UCSC Neanderthal genome browser (https://genome.ucsc.edu/Neandertal/) selecting the full Neanderthal and Denisova assembly tracks.

To determine the rs1805124 allele present in the remaining ancient genomes, blastn searches using the human *SCN5A* nucleotide sequence as query were performed in the available NCBI Sequence Read Archive (SRA) projects: Mal’ta1-PRJNA218466, Kostenki14-PRJEB7618, Paleo eskimo-PRJNA46213, Tyrolean Iceman Otzi-PRJEB2830. The resulting hits were collected and aligned to human *SCN5A* nucleotide sequence and the corresponding position was identified. The analysis of rs1805124 in primates and major mammalian lineages entailed the collection of orthologous *SCN5A* nucleotide sequences (Table S2). If fully annotated sequences were not available in NCBI nucleotide database, sequences were manually assembled using SRA data. For this human *SCN5A* nucleotide sequence was used as query and blastn searches were performed in the corresponding SRA projects (Table S3). The resulting hits were collected and uploaded into Geneious V7.1.9 and assembled using the sequence map to reference tool. Next, sequences were aligned and the homologous position to human rs1805124 was inspected in each species. Additionally, it was further evaluated the polymorphic status of the rs1805124 in primates, viewing which we selected a subset of species encompassing those with SRA data available for several individuals. The Homininae species investigated are listed in Table S3.

## Results

### rs1805124 as a Na_*V*_1.5 genetic modifier

The literature search reporting the variation p.His558Arg, as a genetic modifier of the Na_*V*_1.5, essentially uncovered studies that assessed the electrophysiological impact of the variant using whole cell patch clamp techniques (Table S1).

A total of 17 alterations in Na_*V*_1.5 were reported to be modified by the p.His558Arg variant. More specifically, the presence of an arginine residue in position 558 (Arg558) was described to ameliorate the functional outcome of nine deleterious variants: p.Ser216Leu (rs41276525) (Marangoni et al., 2011), p.Arg282His (rs199473083) (Poelzing et al., 2006), p.Thr512Ile (rs199473118) (Viswanathan, Benson & Balser, 2003), p.Asp1275Asn (rs137854618) (Gui et al., 2010), p.Asp1690Asn (rs1060499900), p.Gly1748Asp (Núñez et al., 2013), p.Met1766Leu (rs752476527) (Ye et al., 2003), p.Val1951Leu (rs41315493) and p.Pro2006Ala (rs45489199) (Shinlapawittayatorn et al., 2011). By contrast, in other studies Arg558 was found to aggravate the functional outcome of the protein product when it co-occurred with the following eight deleterious replacements: p.Arg222Gln (rs45546039) (Cheng et al., 2010), p.Gly400Ala (rs199473106) (Hu et al., 2007), p.Glu161Lys (rs199473062), p.Ala572Asp (rs36210423), p.Pro1298Leu (rs28937319), p.Arg1632His (rs199473286) (Gui et al., 2010), p.Glu1784Lys (rs137854601) (Veltmann et al., 2016) and p.Ile1835Thr (rs45563942) (Cheng et al., 2010).

The presence of Arg558 showed no functional effect on the following nine alterations: p.Leu212Pro, p.Thr220Ile, p.Phe1617del, p.Thr1871Ile, p.Arg878Cys, p.Gly1408Arg, p.Trp1421Ter, p.Lys1578fs/52 and p.Arg1632Ter (Gui et al., 2010).

**Figure 1:**
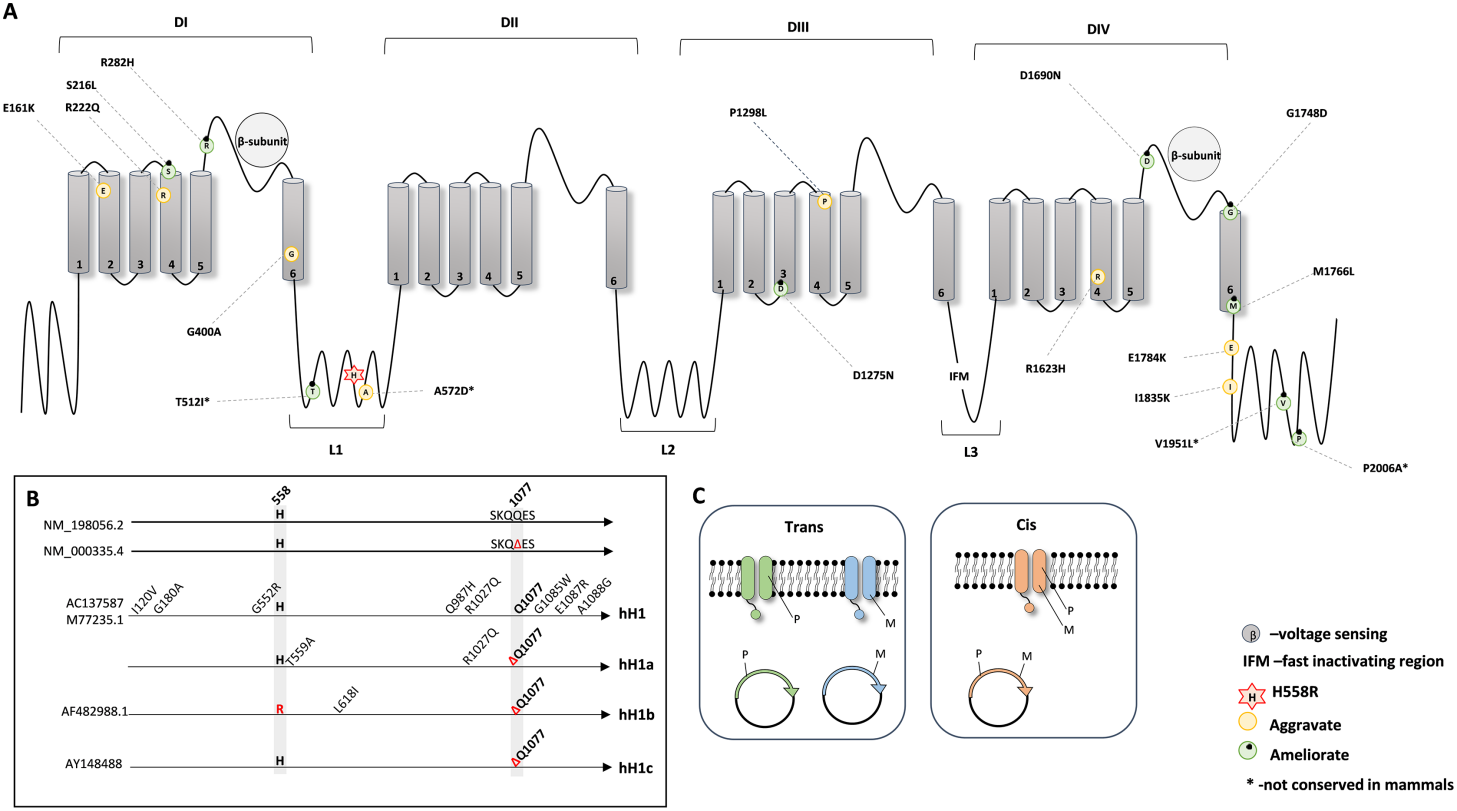
Schematic representation of the NaV1.5 protein. (A) 2D topology of the protein showing the localization of the p.His558Arg variant in Linker 1 (L1) and the localization of the reported deleterious variants. (B) Schematic representation of the genetic backgrounds in which p.His558Arg was expressed and functionally characterized. (C) Illustration of the membrane composition showing cis and trans allelic backgrounds, where P indicates the presence of the polymorphic p.His558Arg variant and M indicates the presence of a deleterious variant.

Next, to investigate the protein position of all the mentioned amino acid substitutions in relation to p.His558Arg, we combined the information from the available crystal structures (PDB: 6UZ3 and 6UZ0) (Jiang et al., 2020) and from UniProt database and plotted a schematic secondary structure of Na_*V*_1.5 showing the location of the deleterious variants modified by p.His558Arg (Fig. 1A).

Na_*V*_1.5 is a transmembrane protein constituted by four domains (DI, DII, DIII, and DIV), all containing six transmembrane segments (S1, S2, S3, S4, S5 and S6), with each domain being linked to the next through cytoplasmic linker segments (L1, L2, and L3) (Balser, 1999; Jiang et al., 2020). While p.His558Arg is localized in linker L1, we found that the majority of the deleterious variants that are positively modulated (compensated) by the Arg558 are located in loops or linker regions, whereas deleterious variants negatively modulated by Arg558 are mostly buried in transmembrane regions but can also locate in loops or linker regions (Fig. 1A).

We further examined the experimental details used for the functional characterization of p.His558Arg combined with different target deleterious variants. Although the accessed data relied on the whole cell patch clamp technique, some critical parameters varied between the original studies. For instance, different genetic backgrounds were used to express and characterize the functional outcome of the p.His558Arg variant in combination with a given co-occurring deleterious variant (Fig. 1B). In humans, the *SCN5A* gene presents two major splice isoforms, namely the neonatal and the adult isoform that among other differences contain a glutamine residue at position 1077 (NM_198056.2) or not (NM_000335.4). The most frequently assayed genetic background to express and characterize functionally the *SCN5A* protein corresponds to the neonatal isoform, despite encoding a protein with the above mentioned amino acid difference comparatively to the adult form. Four distinct clones have been in the patch clamp assays: hH1 (M77235.1 or AC137587), hH1a, hH1b (AF482988.1) and hH1c (AY148488) (Fig. 1B). The hH1 clone, which differs from the reference sequences (NM_000335.4 and NM_198056.2) by eight missense variants, was employed in five studies (Gui et al., 2010; Marangoni et al., 2011; Núñez et al., 2013; Viswanathan, Benson & Balser, 2003; Ye et al., 2003). The clone hH1a, which contains two amino acid differences when compared to the reference sequences was used in two reports (Hu et al., 2007; Ye et al., 2003), the hH1b clone was used in one study (Ye et al., 2003); the hH1c clone, which is identical to the reference sequence NM_000335.4, was elected in four studies (Cheng et al., 2010; Poelzing et al., 2006; Shinlapawittayatorn et al., 2011; Veltmann et al., 2016). In addition to the distinct genetic backgrounds, there are also other confounding factors in different studies (Núñez et al., 2013; Poelzing et al., 2006; Shinlapawittayatorn et al., 2011), namely inconsistency in the investigation of the modulating effect of the Arg558 when expressed together with the target deleterious variant (*cis*) and/or when expressed in its absence (*trans*) (Fig. 1C). Still, despite the non-uniform parameters used to characterize the SCN5A protein, all studies showed a functional modifier effect caused by the simultaneous presence of the p.His558Arg and each deleterious variant in comparison to the corresponding control backgrounds.

### rs1805124 and LD in humans

The allelic distribution of the rs1805124 variant at the 1KGP revealed that the highest frequency of allele C (coding Arg558) was observed in African populations (0.309), followed by American (0.228), South Asian (0.273), European (0.217) and lastly East Asian (0.101) populations (Table S4). Homozygous individuals for the C allele were found in all populations except in two East Asian subpopulations (CHB and KHV, Table S4) and accordingly with the allele frequencies, the African superpopulation showed the highest frequency, reaching 0.106, followed by the South Asian with a frequency of 0.078. We further investigated if the average allelic frequencies of the superpopulations are representative of all populations included within, by conducting a Bayesian one sample t-test where the superpopulation mean allele frequency was assumed as the test value (H0) (Morey et al., 2011; Rouder et al., 2009). Although four populations (ESN, GWD, MSL and IBS) presented mean allelic frequencies not included within the 95% confidence interval, the results from the t-test favoured the null hypothesis, indicating that the superpopulation mean frequencies were indeed good representative values (Table S5).

We further investigated the LD pattern of the deleterious variants previously reported to be modified by the polymorphic rs1805124. Using the LDlink database (Machiela & Chanock, 2015), no evidence was found of LD between rs1805124 (p.His558Arg) and rs199473062 (p.Glu161Lys), rs41276525 (p.Ser216Leu), rs45563942 (p.Ile1835Thr), rs41315493 (p.Val1951Leu) or rs45489199 (p.Pro2006Ala) in all populations analysed. For the remaining variants responsible for disease-associated amino acid replacements modulated by p.His558Arg (identified above), LD data were not available due to their low frequency.

We extended the LD analysis to include other positions within 500 kbp around the site p.His558Arg that have been associated to cardiovascular measurement traits or cardiovascular disease (Table 1). According to the data from GWAS catalogue in EBI (Buniello et al., 2019), six sites were in LD with rs1805124. In detail, the variant rs2051211 (A/C/G forward strand) was found in LD with rs1805124 in African and European populations, being of note that GWAS studies (GCST003844, GCST003598) documented the connection between this variant and alterations in the QRS interval with the G allele being considered the risk allele (Evans et al., 2016). Another variant in LD with rs1805124 was rs3922844 (C/T forward strand), which reportedly was associated to PR interval, QRS duration, P wave duration, and supraventricular ectopy in several independent studies. While the C allele was showed to be the risk allele for PR interval (Smith et al., 2011), QRS duration (Evans et al., 2016; Swenson et al., 2019), and decreased *SCN5A* RNA expression in the human cardiac tissue (Evans et al., 2016), the T allele was the risk allele in supraventricular ectopy (Napier et al., 2018), PR interval (Butler et al., 2012), QT interval (Méndez-Giráldez et al., 2017) and P-wave duration (Christophersen et al., 2017). The LD analysis coherently revealed an association between rs1805124-C and the rs3922844-T in the American, East Asian, European and South Asian populations. A third variant, rs7374004 (A/T forward strand), was associated to increased PR interval (GCST005080) in American, European and South Asian populations (Seyerle et al., 2018). The risk allele was bearing the A nucleotide (Seyerle et al., 2018), which was here shown to be in LD with the rs1805124 C allele. Regarding the fourth variant, rs7374540 (C/A forward strand), the reported risk allele harbouring an A previously associated with atrial fibrillation (Nielsen et al., 2018; Roselli et al., 2018) (GCST006414, GCST006061), was found to co-segregate with the rs1805124 C allele in African and East Asian populations. Also in the case of the rs11710077 (A/T forward strand) linked to QT interval anomalies (Arking et al., 2014; Sotoodehnia et al., 2010), the risk allele T (GCST002500) was here evidenced to be in LD with the rs1805124 C allele. On the other hand, the risk allele C of rs6599222 (C/T forward strand) associated to anomalies in the PR interval (GCST000971, GCST008042) (Smith et al., 2011) was here shown to be in LD with the rs1805124 T allele. LD analysis demonstrated a strong non-random association in allelic segregation (D′ > 0.7) in most of the pairwise polymorphisms analysed, and the rs1805124-C allele was shown to co-segregate with at least four assumed risk alleles, while the rs1805124 T allele was associated to one risk allele (rs6599222) in African populations.

**Table 1:** LD analysis of the rs1805124 variant. Risk allele frequency obtained from 1KGB phase 3, R^2^-measure of correlation of alleles for two genetic variants, were a value of 0 indicates alleles are independent, whereas a value of 1 indicates full correlation. D’ is an indicator of allelic segregation for two genetic variants. D’ values range from 0 to 1 with higher values indicating tight linkage of alleles. Underlined alleles correspond to reported ancestral alleles.

		GWAS trait	Risk allele	Risk allele frequency	R^2^	D′	*p*-value	Correlation with rs1805124 (C-Arg)
American	rs2051211 (A/C/G)	QRS duration	**G**	0.223	0.1579	0.4169	<0.0001	**G**
European					0.1477	0.4201	<0.0001	
American	rs3922844 (C/T)	PR interval, QRS duration, P wave duration, Superventricular ectopy	-	**T**-0.363 **C**-0.637	0.4144	0.8731	<0.0001	**T**
East Asian					0.6884	0.9218	<0.0001	
European					0.5567	0.9094	<0.0001	
South Asian					0.4890	0.7263	<0.0001	
American	rs7374004 (A/T)	PR interval	**A**	0.693	0.1437	0.9632	<0.0001	**A**
European					0.1941	0.9577	<0.0001	
South Asian					0.2232	0.9267	<0.0001	
African	rs7374540 (C/A)	Atrial fibrillation	**A**	0.447	0.2457	0.7008	<0.0001	**A**
East Asian					0.1074	0.9256	<0.0001	
African	rs6599222 (C/T)	PR interval	**C**	0.155	0.1165	0.9559	<0.0001	**T**
African	rs11710077 (A/T)	QT interval & QRS duration	**T**	0.144	0.1808	0.9134	<0.0001	**T**
American					0.2818	0.621	<0.0001	
East Asian					0.3982	0.8491	<0.0001	
European					0.2027	0.4625	<0.0001	
South Asian					0.2584	0.5886	<0.0001	

**Figure 2:**
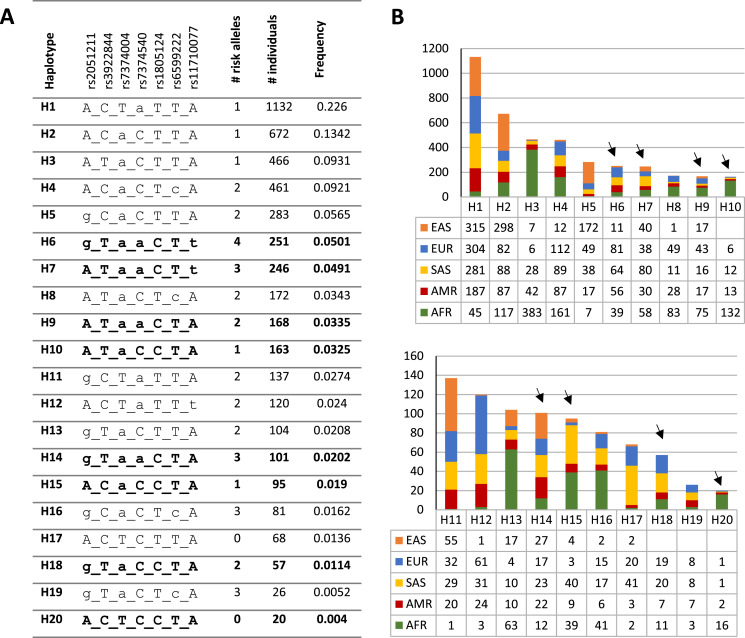
Haplotypic structure. (A) Haplotypic analysis based on the 1KGP data obtained through combination of variants rs2051211, rs3922844, rs7374004, rs7374540, rs6599222 and rs11710077 found to be in LD with rs1805124. Frequency and count values refer to the sum off all populations; lower case letters correspond to risk alleles; bold haplotypes carry the rs1805124-C allele. Only the top 20 most frequent haplotypes are represented (full list of haplotypes in Table S6). (B) Frequency distribution of each haplotype in all populations analysed. Black arrowheads indicate haplotypes carrying the rs1805124-C allele.

To investigate the distribution and frequency of the haplotypes encompassing rs1805124, the coordinates of the genomic location of each variant were used to indicate the different alleles in the haplotype according to the following order: rs2051211 (A/C/G), rs3922844 (C/T), rs7374004 (A/T), rs7374540 (A/T), rs1805124 (C/T), rs6599222 (C/T) and rs11710077 (A/T). Out a total of 50 haplotypes found in the whole human population (Table S6), the most frequent is H1 ACTaTTA (lower case letters correspond to risk alleles) with a frequency of 0.226, which is a haplotype that carries one risk allele at rs7374540 (Figs. 2A and 2B and Table S6). The following four most frequent haplotypes (H2–H5), all presenting the T allele in the position rs1805124, carried a maximum of two risk alleles. The most common (0.0501) haplotype carrying the C allele at the rs1805124 is H6 (gTaaCTt) which exhibits all the alleles previously found to be in LD (Figs. 2A and 2B, Table 2), thus bearing four risk alleles. H7 displays a similar frequency (0.0491) and harbours three risk alleles including the C allele at rs1805124. Only two haplotypes in the 1GKP, H17 and H20, present at the low frequencies of 0.0136 and 0.004 respectively, both carrying the rs1805124 C allele, were destitute of any risk alleles.

**Table 2:** LD in human ancient genomes.

Sample	rs2051211 A/C/G	rs3922844 T/C	rs7374004 A/T	rs7374540 C/A	rs1805124 C/T	rs6599222 T/C	rs11710077 A/T	Years before present (BP)
*Homo sapiens*	G	T	A	A	**C**	T	T	
Paleo Eskimo	A	–	A	–	**T**	–	A	~4,000
Tyrolean Iceman	–	–	–	–	**T**	–	–	~5,300
Mal´ta1	–	–	–	–	**T**	–	–	~23,891–34,423
Kostenki14	–	–	–	–	**T**	–	–	~36,262–38,684
Vi33.16	–	–	–	–	**C**	–	–	~38,310
Vi33.26	–	–	A	–	**C**	–	A	~44,450
Denisova	A	T	A	A	**C**	T	A	~1,220,000–195,000

### rs1805124 in ancient human genomes

The observed high frequency of the derived allele of rs1805124 (His558) instigated us to investigate the evolutionary history of this polymorphism in archaic humans, primates and major mammalian lineages. We began by exploring human archaic genomes including the Altai Neanderthal (Prüfer et al., 2014), Vindija Neanderthal (Green et al., 2010), Siberian Denisovans (Meyer et al., 2012), Mal’ta1 (Raghavan et al., 2014), Kostenki14, Paleo Eskimo (Rasmussen et al., 2010) Tyrolean Iceman Otzi (Keller et al., 2012).

**Figure 3:**
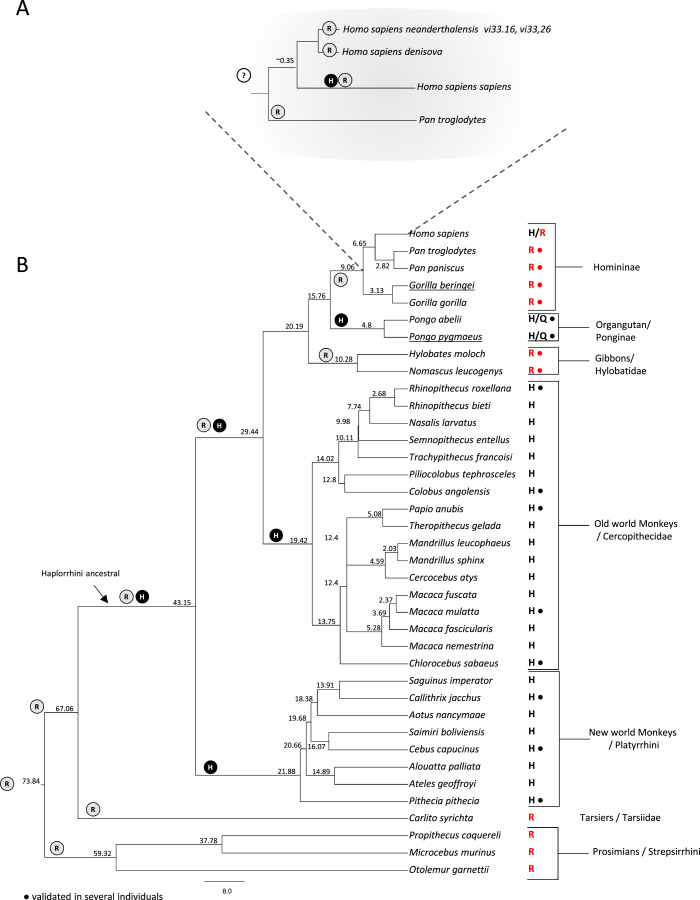
Investigation of the rs1805124 allelic status in primates. Primate phylogenetic species tree calculated in www.Timetree.org (Kumar et al., 2017). Values at tree nodes indicate approximate time of lineage divergence. The predicted ancestral state of position 558 is represented by circled H (histidine) or R (arginine). The extant state of position 558 is indicated for each specie analysed. Dots indicate the analysis of several independent individuals of a given species. (A) Detailed view of the Homininae lineage. (B) Full view of the primate species analysed.

Our analyses revealed that Neanderthal genomes from vi33.16 and vi33.26 and the Denisovan genome all have the C allele in the corresponding position of rs1805124 (Table 2, Fig. 3A). No read coverage in this region was available for the Neanderthals Felhofer (Feld1) Mezmaiskaya (Mez1), El Sidrón (Sid1253) and Vindija (Vi33.25) which was anticipated due to the low coverage of these genomes. In contrast, Kostenki14, Mal’ta1, Tyrolean Iceman and Paleo Eskimo genomes showed the T allele in the rs1805124 position. In the case of the Denisova we were able to obtain a full haplotype involving the variants linked to rs14805124, which revealed to correspond to haplotype H9.

### Evolutionary analysis of the rs1805124 variant in mammals

To gain insight into the evolutionary trajectory of the rs1805124 variant, a comparative analysis was performed in a total of 38 primates including great apes (*Homo sapiens, Pan troglodytes, Pan paniscus, Gorilla beringei, Gorilla gorilla, Pongo abelii* and *Pongo pygmaeus*) gibbons, Old World monkeys, New World monkeys, Tarsiers and Prosimians (Fig. 3). Concerning the *H. sapiens* closest extant relatives, chimpanzees (*P. troglodytes* and *P. paniscus*) and gorillas (*Gorilla beringei, Gorilla gorilla*), all presented the C allele in the position corresponding to rs1805124. To assess whether or not these species are also polymorphic, we analysed several individuals for each species (Table S3), ending up with 30 chimpanzees from three subspecies (*Pan troglodytes ellioti, Pan troglodytes troglodytes, Pan troglodytes schweinfurthii*); 13 bonobos (*Pan pansicus*) and 19 gorillas from two species and three subspecies (*Gorilla gorilla gorilla, Gorilla gorilla diehli* and *Gorilla beringei graueri*). All samples for which sequence reads were collected in this region presented the rs1805124-C allele, a strong indication that this position is fixed in these species. In the orangutans examined (*Pongo pygmaeus* and *Pongo abelii*) the analysis of 10 specimens showed that all SRA reads collected presented the T allele, but three reads (one from *Pongo abelii* and two from *Pongo pygmaeus*) showed a glutamine (Q) at position 558. All gibbons show the rs1805124 C allele only.

Regarding the Old and New World monkeys, all species analysed presented the T allele (histidine). To ascertain the polymorphic status we examined SRA reads from a representative set of species from this group, namely *Rhinopithecus roxellana, Colobus angolensis, Papio anubis, Macaca mulatta, Chlorocebus sabaeus, Callithrix jacchus, Cebus capucinus* and *Pithecia pithecia* but no evidence was found of intraspecific polymorphism both in Old and New World monkeys. Lastly, the analysis of Tarsiers and Prosimians showed that these species presented the C allele (arginine).

The majority of the remaining mammalian groups (Rodentia, Lagomorpha, Artiodactyla, Cetacea, Perissodactyla, Chiroptera, Afrotheria, and Marsupialia), show an arginine in position 558 (Table S2). The two exceptions are the rat (*Rattus norvegicus*) which was polymorphic and the giant panda (*Ailuropoda melanoleuca*). Overall, after examining the position corresponding to the human rs1805124 in mammals we got to know that this position patchily varies between the C and the T allele in different lineages. Since the C allele (arginine) is the one more frequently found, most likely represents the ancestral allele.

## Discussion

The effect of *cis*-related genetic heterogeneity can underlie incomplete penetrance, allelic imbalance, and compensatory effects among other phenomena (Cooper et al., 2013; Lopes-Marques et al., 2021; Serrano et al., 2021). Several studies have shown that the rs1805124 variant is able to act as a genetic modifier of many *SCN5A* disease-associated variants (*e.g*. (Matsumura et al., 2017; Shinlapawittayatorn et al., 2011; Viswanathan, Benson & Balser, 2003)). Yet, the mechanism through which this variant modulates the effect of other variants still remains far from fully understood. The analysis of literature regarding rs1805124 has provided evidence for at least two *modus operandi* through which the rs1805124 may modulate the functional outcome of Na_*V*_1.5.

One mechanism implies the full or partial functional rescue of Na_*V*_1.5 from deleterious variants that interfere with protein folding and cell trafficking, characterized by reduced or absent *I*_Na_ currents. It was demonstrated that the rs1805124 variant fully rescued the functional impairment caused by p.Arg282His (Poelzing et al., 2006) when expressed in *trans*, while in *cis* it was able to attenuate the effect of p.Ser216Leu, p.Asp1275Asn, p.Asp1690Asn and p.Met1766Leu (Gui et al., 2010; Marangoni et al., 2011; Núñez et al., 2013; Ye et al., 2003). According to some studies, since *SCN5A* proteins interact within the cell, the linker region where p.His558Arg is situated may play a role in their stabilization, folding and cell trafficking (Gui et al., 2010; Marangoni et al., 2011; Núñez et al., 2013; Poelzing et al., 2006; Shinlapawittayatorn et al., 2011; Ye et al., 2003).

A second mechanism by which rs1805124 may modulates Na_*V*_1.5 function is through regulation of mRNA expression. A previous work on human atrial cardiac tissue samples showed that the rs1805124-T allele (His558) drove higher mRNA expression levels in comparison to samples carrying the C allele (Arg558), possibly by modulating DNA methylation of *SCN5A* promoters (Matsumura et al., 2017). It is of note that the codon for arginine 558 is a CGC which may make this triplet more prone to methylation (Cooper et al., 2010). Also, the linker region (L1-Fig. 1A) in which the p.His558Arg variant is localized was reported to be a hot-spot for arginine methylation (Beltran-Alvarez et al., 2013; Beltran-Alvarez, Pagans & Brugada, 2011) with at least three arginine residues (Arg513, Arg526, and Arg680) in this region being known to be modified by methylation. The amino acid replacements p.Arg526His and p.Arg680His have been linked to Brugada syndrome and sudden infant death syndrome, respectively (Kapplinger et al., 2010; Wang et al., 2007). To this date, the studies regarding post-translational modification of Na_*V*_1.5 proteins examined sequences containing a histidine at position 558.

In addition to known deleterious variants, the rs1805124 also influences the functional outcome of alternative splice isoforms of *SCN5A* (p.Gln1077del) and missense variants reported as benign namely p.Ala572Asp (rs36210423). In the case of the p.Ala572Asp, functional characterization in the hH1C background showed no differences to wild-type combined with His558. However, when the p.Ala572Asp was combined with Arg558, the functional outcome was negatively affected (Tester et al., 2010). Regarding the p.Gln1077del alternative splice isoform, the functional outcome of SCN5A carrying the rs1805124-C allele was dependent of the genetic background (p.Gln1007del or Gln1077) in which this variant was characterized (Tester et al., 2010). In the case of the p.Gln1077del the presence of Arg558 was shown to aggravate the functional effect in the hH1 (AC1377587) background; however, in the different background hH1c p.Gln1077del no functional modelling effect of the Arg558 allele become apparent (Tester et al., 2010). The aggravation observed on the hH1 background could be due to the cumulative effect of eight missense variants observed in this clone in relation to the reference sequence (Fig. 1B), while the hH1c clone presents no differences from the reference *SCN5A* sequence (NM_000335.4).

To identify additional players underlining the modulating effect of rs1805124 variant we performed LD analysis, permitting to detect that rs1805124 is in LD with to six other polymorphic sites which were also associated with cardiac traits in GWAS studies. Two of them, rs3922844 and rs11710077, have been shown to play a role in *SCN5A* expression (Evans et al., 2016; Kapoor et al., 2019). The rs3922844-C allele, previously associated with reduced levels of *SCN5A* expression (Evans et al., 2016), was here shown to co-segregate with the rs1805124-T allele (histidine), being thus possible that the levels of pathogenicity of rs3922844-C allele depend on the rs1805124 allele with which it segregates. This might also explain that the reason why both nucleotides at rs3922844 had been considered as risk alleles in distinct GWAS. In addition to rs3922844, a second variant may also be involved in the modulation of *SCN5A* expression, namely rs11710077, which is located in a *cis*-regulatory element of intron 17 (Kapoor et al., 2019). The reported risk allele at rs11710077 co-segregates with the rs1805124-C allele. Furthermore, rs1805124 was shown to be in LD with four risk alleles at rs2051211, rs7374004, rs7374540 and rs11710077. Accordingly, haplotype analysis showed that the most frequent haplotype carrying a C allele was H6 in which all four risk alleles are present.

The reconstruction of the evolutionary history of this variant supported that the ancestral allele in mammals is rs1805124-C, which encodes arginine. A total of 48 non-primate species covering all major lineages harbour the C allele, with the exception of *Rattus norvergicus* in which, similarly to humans, rs1805124 is polymorphic, and *Ailuropoda melanoleuca* that only shows the T allele.

The majority of primate species had fixed either the C or the T allele, with the exception of humans who are polymorphic for rs1805124. The analysis of ancient human genomes showed that both the Neanderthals and the Denisova shared the C allele, similarly to humans’ closest extant relatives, chimpanzees and gorillas. This observation lead us to inquire why is the C allele less frequent in human populations. Aside from the functional modulation triggered by the p.His558Arg reported in works performing *in vitro* functional characterization of the Na_*V*_1.5 (Cheng et al., 2010; Gui et al., 2010; Marangoni et al., 2011; Poelzing et al., 2006; Shinlapawittayatorn et al., 2011), we ascertained that an aggregation of risk alleles with rs1805124-C may account for the reduced frequency of this allele in humans. Seemingly, this is supported by other studies which have found that p.His558Arg variant is more frequent in patients with Brugada syndrome (Matsumura et al., 2017) or Lone atrial fibrillation (Chen et al., 2011; Chen et al., 2007), among whom no additional missense or splice site deleterious variants in *SCN5A* were detected in comparison to controls.

## Conclusions

The p.His558Arg variant poses a unique case of complexity in the modulation of Na_*V*_1.5 activity, as it has been reported to act as a genetic modifier “for good and bad”. In this study, the reanalysis of published data allowed us to predict that the modulation effect of p.His558Arg is dependent not only on whether it is in *cis* or in *trans* with the linked deleterious variant, but also on the set of linked polymorphic variants to which it is anchored. Importantly, six of these variants have been previously associated with cardiac phenotypes in GWAS, being, therefore, good candidates for the discrepancies observed on the functional effect of p.His558Arg. In this context, our study is providing a more comprehensive picture of its effect and an updated contribution to genetics of cardiac disorders. We have also demonstrated that the His558 (T allele) is the most frequent allele found in all human populations analysed, although the Arg558 (C allele) is the most frequent allele found in non-human primate species which lead us to conclude that Arg558 must be the primate ancestral configuration. Additionally, the observation that the His558 allele is also present in the Prosimians, makes it possible that this allele has prevailed during the mammalian evolutionary history. The importance of genetic interactions between variants in this or other genes should deserve further attention in a desirably near future, as the influence of such interactions in the road that leads to phenotypic outcomes might be stronger than currently anticipated.

## Supplemental Information

10.7717/peerj.13913/supp-1 Supplemental Information 1 Supplementary material.

## Additional Information and Declarations

### Data Availability

The following information was supplied regarding data availability: Raw data are available as a Supplemental File.
