# Supplementary material for: Complex interactions between p.His558Arg and linked variants in the sodium voltage-gated channel alpha subunit 5 (NaV1.5)
Source: PeerJ. 2022 Aug 17;10:e13913. doi: 10.7717/peerj.13913 (PMC9392453; doi:10.7717/peerj.13913)
Supplement: Supplemental Information 1 [file peerj-10-13913-s001.pdf]

Complex interactions between p.His558Arg and linked variants in the Sodium Voltage-Gated Channel Alpha Subunit 5 (Na_V1.5)

Mónica Lopes-Marques[1,2,3*]; Raquel M. Silva[4], Catarina Serrano[1,2,3], Verónica Gomes[1,2], Ana Rita Cardoso[1,2,3], Maria J. Prata[1,2,3], António Amorim[1,2,3], Luísa Azevedo[1,2,3*]

[1] i3S- Instituto de Investigação e Inovação em Saúde, Universidade do Porto, Population Genetics and Evolution Group, Rua Alfredo Allen 208, 4200-135 Porto, Portugal

[2] IPATIMUP-Institute of Molecular Pathology and Immunology, University of Porto, Rua Júlio Amaral de Carvalho 45, 4200-135 Porto, Portugal

[3] Department of Biology, Faculty of Sciences of the University of Porto, Rua do Campo Alegre, s/n, 4169-007 Porto, Portugal

[4] Universidade Católica Portuguesa, Faculdade de Medicina Dentária, Center for Interdisciplinary Research in Health (CIIS), Estrada da Circunvalação, 3504-505 Viseu, Portugal

Table S1. Summary of the collected data from 14 studies regarding the functional impact in patch clamp assays of p.His558Arg. Exp: expression system used in the functional characterization, cis- both deleterious and polymorphic variants are localized in the same construct, trans- deleterious variant and polymorphism are localized in distinct constructs and co-expressed, * Not deleterious variants - A572D, is considered a polymorphism and is found in the general population; also Q1077 and Q1077del corresponds to alternative splice isoform of *SCN5A*. ● AC1377587 sequence associated to this accession number is not available in NCBI. Wt-wild-type.

H558R	Mutation	Exp.	Background	Observations	Outcome	Ref
H	E161K<br>rs199473062	*cis*	M77235	Loss of function (incomplete penetrance)		PMID: 20384651
R	E161K	*cis*	M77235	Steady state inactivation shifted towards hyperpolarizing potentials	Aggravate	
H	S216L<br>rs41276525	*cis*	M77235 (hH1)	Decrease in I_{Na} and misfolding and reduced membrane localization	-	PMID: 21705349
R	S216L	*cis*	M77235 (hH1)	Recovery of I_{Na} , delayed inactivation recovery	Ameliorate	
H	R222Q+Q1007del	*cis*	AY148488	I_{Na} density normal, alterations in the activation and inactivation negative shift	-	PMID: 21167004
R	R222Q+Q1007del	*cis*	AY148488	35% reduction in I_{Na} density, alterations in the activation and inactivation negative shift, slower recovery from inactivation	Aggravate in the Q1077 del	
H	R222Q<br>282h	*cis*	AC1377587*	I_{Na} density normal, alterations in the activation and inactivation negative shift	-	
R	R222Q	*cis*	AC1377587*	I_{Na} density normal, alterations in the activation and inactivation negative shift	-	
H	R282H	*trans*	NM 198056	Reduced ~58% whole cell currents	-	PMID: 16864729
H	Wt		NM 198056			
H	R282H	*trans*	NM 198056	Normal current	Full rescue through trafficking	
**R**	Wt		NM 198056			
**R**	R282H	*trans*	NM 198056	Reduced ~48% whole cell currents	-	
H	Wt		NM 198056			
H	Wt	*trans*	NM 198056	Normal current	Normal	
R	Wt		NM 198056			
H	R282H	*cis*	NM 198056	No current	-	
R	R282H	*cis*	NM 198056	No current	-	
H	G400A		hH1a	Reduced peak current		PMID: 17675083
R	G400A		hH1a	Further reduced peak current	Aggravate	
H	T512I	*cis*	M77235 (hH1)	Increased development of slow inactivation	-	PMID: 12569159
R	T512I	*cis*	M77235 (hH1)	Enhanced slow activation	Ameliorate	
H	*A572 + Q1077del		hH1C	normal		PMID: 20403459
R	*A572+ Q1077del			normal		
H	*A572D+ Q1077del		17	Peak current equal to Wt		
R	*A572D+ Q1077del			Not significant lower peak current, increase in late persistent current, significant negative shift in the steady states inactivation	Aggravate Double hit mutation linked to R558	
H	*Q1077		AC1377587●	Reduced current (not significant)	Normal	
R	*Q1077		AC1377587●	Little or No I_{Na} density??	Aggravate	
H	*Q1077del		AY148488	Normal currents	Normal	
R	*Q1077del		AY148488	Normal currents	Normal	
R	*Q1077	*trans*	AC1377587●	Normal currents	Normal	
R	*		AY148488			
H	D1275N	*cis*	M77235 (hH1)	Reduced peak current		PMID: 20384651
R	D1275N	*cis*	M77235 (hH1)	2 fold peak increase in peak current comparable to Wt, Hyperpolarizing potential, negative shifts of the midinactivation	Ameliorate Through intracellular trafficking	
H	D1632H	*cis*	M77235 (hH1)	Loss of function		
R	D1632H	*cis*	M77235 (hH1)	Hyperpolarizing potential, negative shifts of the mid inactivation	Aggravate	

Continues next page

H558R	Mutation	*Exp.*	Background	Observations	Outcome	Ref
H	P1298L	*cis*				PMID: 20384651
R	P1298L	*cis*		Hyperpolarizing potential, negative shifts of the mid inactivation	Aggravate	
H	-	*cis*	M77235 (hH1)	Normal peak currents		PMID: 23085483
H	D1690N	*cis*	M77235 (hH1)	Reduced peak currents, defective trafficking to the membrane		
H	G1748D	*cis*	M77235 (hH1)	Very low peak currents, defective trafficking to the membrane		
H	-	*trans*	M77235 (hH1)	30% reduced peak current		
H	D1690N		M77235 (hH1)			
H	-	*trans*	M77235 (hH1)	15% reduced peak current		
H	G1748D		M77235 (hH1)			
**R**	-	*trans*	M77235 (hH1)	Reduced Current		
H	D1690N		M77235 (hH1)			
**R**	-	*trans*	M77235 (hH1)	Reduced current		
H	G1748D		M77235 (hH1)			
**R**	D1690N	*cis*	M77235 (hH1)	Restoration of Wt currents, correct trafficking to the membrane	Ameliorate	
**R**	G1748D	*cis*	M77235 (hH1)	Very low peak currents		
R	M1766L		hH1b	Normal currents and cell traficking	Ameliorate	PMID: 12454206
H	M1766L	*cis*	M77235 (hH1)	Reduced currents		
H	M1766L	*cis*	hH1a	Reduced currents		
R	M1766L	*cis*	hH1a	Normal currents and cell trafficking restores	ameliorate	
H	E1784K	*trans*	hH1c	Reduced currents		PMID: 27381756
H	-		hH1c			
H	E1784K	*trans*	hH1c	More prominent reduced currents	Aggravate	
R	-		hH1c			
H	I1835T		AC1377587●	I_{Na} density normal, late I_{Na} normal	Normal	PMID: 21167004
R	I1835T		AC1377587●	I_{Na} density normal, late I_{Na} normal, negative shift in inactivation, normal recovery from inactivation	Aggravate	
H	I1835T+Q1007del		AY148488	No significant differences	Normal	
R	I1835T+Q1007del		AY148488	30% reduction in the I_{Na} density, negative shift in inactivation, slower recovery from inactivation	Aggravate ++ in a Q1007del	
H	P2006A		NM 198056	Increased persistent sodium currents, and +10mV shift in voltage dependence of steady state in activation, faster recovery from inactivation		PMID: 21109022
R	P2006A		NM 198056	Sodium currents recovered values comparable with Wt	Ameliorate	
H	P2006A	*trans*	NM 198056	Sodium currents recovered values comparable with Wt	Ameliorate	
R	P2006A					
R	-	*trans*	NM 198056	Sodium currents recovered values comparable with Wt	Ameliorate	
R	P2006A					
H	-	*trans*	NM 198056	+9mV shift in steady state inactivation	-	
R	V1951L		NM 198056	Recovery to Wt values	Ameliorate	

Table S2. Accession numbers of *SCN5A* sequences for species with no annotated *SCN5A*. Gene manual annotation was performed and scaffold numbers where *SCN5A* was identified are provided. In the case of species with no *SCN5A* annotation and no genome assembly, SRA projects were searched and project numbers are indicated in Table S3.

		Species	Order	Accession number SCN5A Neonatal	Allele
**1**	HSA	*Homo sapiens*	Primate-Hominoidae	NM_198056.2	H/R
**2**	PPA	*Pan paniscus*	Primate-Hominoidae	XM_008951545.1	R
**3**	PTR	*Pan troglodytes*	Primate-Hominoidae	XM_016940745.2	R
**4**	GBE	*Gorilla beringei*	Primate-Hominoidae	SRA	R
**5**	GGO	*Gorilla gorilla*	Primate-Hominoidae	XM_019024737.2	R
**6**	PAB	*Pongo abelii*	Primate-Hominoidae	XM_024245225.1	H/Q
**7**	PPY	*Pongo pygmaeus*	Primate-Hominoidae	SRA	H/Q
**8**	HMO	*Hylobates moloch*	Primate-Hominoidae	XM_032162037.1	R
**9**	NLE	*Nomascus leucogenys*	Primate-Hominoidae	XM_030812100.1	R
**10**	RBI	*Rhinopithecus bieti*	Primate- Haplorrhini-Cercopithecoidea	XM_017849653.1	H
**11**	RRO	*Rhinopithecus roxellana*	Primate- Haplorrhini-Cercopithecoidea	XM_010371496.2	H
**12**	NLA	*Nasalis larvatus*	Primate- Haplorrhini-Cercopithecoidea	SRA	H
**13**	SEM	*Semnopithecus entellus*	Primate- Haplorrhini-Cercopithecoidea	SRA	H
**14**	TFR	*Trachypithecus francoisi*	Primate- Haplorrhini-Cercopithecoidea	XM_033212073.1	H
**15**	PTE	*Piliocolobus tephrosceles*	Primate-Haplorrhini-Cercopithecoidea	XM_023231462.1	H
**16**	CAN	*Colobus angolensis*	Primate- Haplorrhini-Cercopithecoidea	XM_011953304.1	H
**17**	PAN	*Papio anubis*	Primate- Haplorrhini-Cercopithecoidea	XM_009201170.3	H
**18**	TGE	*Theropithecus gelada*	Primate- Haplorrhini-Cercopithecoidea	XM_025375222.1	H
**19**	MLE	*Mandrillus leucophaeus*	Primate- Haplorrhini-Cercopithecoidea	XM_011999484.1	H
**20**	MSP	*Mandrillus sphinx*	Primate- Haplorrhini-Cercopithecoidea	SRA	H
**21**	CAT	*Cercocebus atys*	Primate- Haplorrhini-Cercopithecoidea	XM_012034251.1	H
**22**	MFU	*Macaca fuscata*	Primate- Haplorrhini-Cercopithecoidea	SRA	H
**23**	MMUL	*Macaca mulatta*	Primate- Haplorrhini-Cercopithecoidea	XM_015131334.1	H
**24**	MNE	*Macaca nemestrina*	Primate- Haplorrhini-Cercopithecoidea	XM_011738773.2	H
**25**	MFA	*Macaca fascicularis*	Primate- Haplorrhini-Cercopithecoidea	XM_005546683.2	H
**26**	CSA	*Chlorocebus sabaeus*	Primate- Haplorrhini-Cercopithecoidea	XM_007971735.1	H
**27**	SIM	*Saguinus imperator*	Primate- Haplorrhini-Platyrrhini	SRA	H
**28**	CJA	Callithrix jacchus	Primate- Haplorrhini-Platyrrhini	XM_017965958.1	H
**29**	ANA	*Aotus nancymaae*	Primate- Haplorrhini-Platyrrhini	XM_021666569.1	H
**30**	SBO	*Saimiri boliviensis boliviensis*	Primate- Haplorrhini-Platyrrhini	XM_010341492.1	H
**31**	CCA	*Cebus capucinus imitator*	Primate- Haplorrhini-Platyrrhini	XM_017508376.1	H
**32**	APA	*Alouatta palliata*	Primate- Haplorrhini-Platyrrhini	PVKV010013273.1<br>PVKV010001690.1	H
**33**	AGE	*Ateles geoffroyi*	Primate- Haplorrhini-Platyrrhini	PVHS01012057.1<br>PVHS01040726.1	H
**34**	PPI	Pithecia pithecia	Primate- Haplorrhini-Platyrrhini	PVIP01001086.1	H
**35**	CSY	Carlito syrichta	Primate- Haplorrhini- Tarsiidae	XM_008053200.2	R
**36**	OGA	*Otolemur garnettii*	Primate- Strepsirrhini- Lorisiformes	XM_023515918.1	R
**37**	MMUR	*Microcebus murinus*	Primate- Strepsirrhini- Lemuriformes	XM_012756399.1	R
**38**	PCO	*Propithecus coquereli*	Primate- Strepsirrhini- Lemuriformes	XM_012643255.1	R
**39**	NGA	*Nannospalax galili*	Rodentia-Myomorpha	XM_008836179.1	R
**40**	MMU	*Mus musculus*	Rodentia-Myomorpha	NM_001253860.1	R
**41**	RNO	*Rattus norvegicus*	Rodentia-Myomorpha	NM_013125.2	H/R
**42**	CGR	*Cricetulus griseus*	Rodentia-Myomorpha	XM_035444055.1	R
**43**	MOC	*Microtus ochrogaster*	Rodentia-Myomorpha	XM_005348090.3	R
**44**	PMA	*Peromyscus maniculatus*	Rodentia-Myomorpha	XM_006986726.2	R
**45**	MMA	*Marmota marmota marmota*	Rodentia-Sciuromorpha	XM_015489475.1	R
**46**	ITR	*Ictidomys tridecemlineatus*	Rodentia-Sciuromorpha	XM_005317377.2	R
**47**	CPO	*Cavia porcellus*	Rodentia-Hystricomorpha	XM_005001107.2	R
**48**	HGL	*Heterocephalus glaber*	Rodentia-Hystricomorpha	XM_004835238.2	R
**49**	ODE	*Octodon degus*	Rodentia-Hystricomorpha	XM_004642153.2	R
**50**	CLA	*Chinchilla lanigera*	Rodentia-Hystricomorpha	XM_005386578.2	R
**51**	OCU	*Oryctolagus cuniculus*	Lagomorpha	XM_017340134.1	R
**52**	OPR	*Ochotona princeps*	Lagomorpha	XM_017340134.1	R
**53**	SSC	*Sus scrofa*	Cetartiodactyla- Suina	XM_021071676.1	R
**54**	CDR	*Camelus dromedarius*	Cetartiodactyla- Camelidae	XM_010974813.1	R
**55**	CFE	*Camelus ferus*	Cetartiodactyla- Camelidae	XM_032459195.1	R
**56**	BTA	*Bos taurus*	Cetartiodactyla- Ruminantia-Bovinae	NM_174458.2	R
**57**	BMU	*Bos mutus*	Cetartiodactyla- Ruminantia-Bovinae	XM_005901131.1	R
**58**	OAR	*Ovis aries*	Cetartiodactyla- Ruminantia-Caprinae	XM_012140142.2	R
**59**	CHI	*Capra hircus*	Cetartiodactyla- Ruminantia-Caprinae	XM_018038398.1	R

**60**	OOR	*Orcinus orca*	Cetartiodactyla-Cetacea-Odontoceti	XM_004277836.1	R
**61**	LVE	*Lipotes vexillifer*	Cetartiodactyla-Cetacea-Odontoceti	XM_007468130.1	R
**62**	PCA	*Physeter catodon*	Cetartiodactyla-Cetacea-Mysticeti	XM_007110496.2	R
**63**	BAC	*Balaenoptera acutorostrata*	Cetartiodactyla-Cetacea-Mysticeti	XM_007191459.1	R
**64**	CSI	*Ceratotherium simum simum*	Perissodactyla-Rhinoceratidae	XM_014790663.1	R
**65**	ECA	*Equus caballus*	Perissodactyla-Equidea	XM_023619740.1	R
**66**	ORO	*Odobenus rosmarus divergens*	Carnivora -Caniformia	XM_012566600.1	R
**67**	CLU	*Canis lupus familiaris*	Carnivora -Caniformia	NM_001002994.1	R
**68**	MFU	*Mustela putorius furo*	Carnivora -Caniformia	XM_004759336.2	R
**69**	AME	*Ailuropoda melanoleuca*	Carnivora -Caniformia	XM_034662224.1	H
**70**	UMA	*Ursus maritimus*	Carnivora -Caniformia	XM_008710149.1	R
**71**	AJU	*Acinonyx jubatus*	Carnivora -Feliformia	XM_027040708.1	R
**72**	PPAR	*Panthera pardus*	Carnivora -Feliformia	XM_019418729.1	R
**73**	FCA	*Felis catus*	Carnivora -Feliformia	XM_023260721.1	R
**74**	DRO	*Desmodus rotundus*	Chiroptera	XM_024565669.1	R
**75**	HAR	*Hipposideros armiger*	Chiroptera	XM_019662785.1	R
**76**	MNA	*Miniopterus natalensis*	Chiroptera	XM_016196168.1	R
**77**	TCH	*Tupaia chinensis*	Scandentia	XM_006161350.2	R
**78**	GVA	*Galeopterus variegatus*	Dermoptera	XM_008567166.1	R
**79**	LAF	*Loxodonta africana*	Afrotheria- Proboscidea	XM_023554825.1	R
**80**	CAS	*Chrysochloris asiatica*	Afrotheria-Afrosoricida	XM_006869133.1	R
**81**	OAF	*Orycteropus afer afer*	Afrotheria-Tubulidentata	XM_007945712.1	R
**82**	CCR	*Condylura cristata*	Insectivora-Talpidae	XM_004676302.2	R
**83**	EEU	*Erinaceus europaeus*	Insectivora- Erinaceomorpha	XM_007517226.2	R
**84**	MDO	*Monodelphis domestica*	Marsupialia	NM_001246327.1	R

Table S3. SRA projects searched to determine the polymorphic status at position 558 of the *SCN5A* gene in other primate species. * indicates that project was searched and no hit overlapping the H558R region was retrieved.

Species	Project	SRX	Sample	Origin	Observations	Allele
Pan troglodytes schweinfurthii	PRJNA189439	SRX237524	Bwanbale	Wild Born	Eastern	R
		SRX237527	Kidongo	Wild Born	Eastern	R
		SRX237541	Nakuu	Wild Born	Eastern	R
	PRJEB2482	ERX012404	Cindy	Chimpanzee Sanctuary		R
		ERX012403	Sally	Chimpanzee Sanctuary		R
		ERX012402	Nakuu	Chimpanzee Sanctuary		*
		ERX012398	Katie	Chimpanzee Sanctuary		*
		ERX012395	Kidogo	Chimpanzee Sanctuary		R
		ERX012394	Kazakuhire	Chimpanzee Sanctuary		*
		ERX012393	Becky	Chimpanzee Sanctuary		R
Pan troglodytes troglodytes	PRJNA189439	SRX243446	Donald	Captive Born	Hybrid	R
		SRX243487	Jimmie	Wild Born	Western	R
		SRX243489	Vailllant	Wild Born	Central	R
		SRX243491	Doris	Wild Born	Central	*
		SRX243493	Julie	Wild Born	Central	R
		SRX243499	Koby	Wild Born	Western	R
		SRX243495	Clara	Wild Born	Central	R
		SRX243448<br>SRX243449	Bosco	Wild Born	Western	R
	PRJEB2482	ERX012401	Marcelle	Tchimpounga Chimp rehabilitation center		R
		ERX012397	Bayokele	Tchimpounga Chimp rehabilitation center		R
		ERX012392	Golfi	Tchimpounga Chimp rehabilitation center		*
		ERX012391	Gao	Tchimpounga Chimp rehabilitation center		R
		ERX012390	FanTuek	Tchimpounga Chimp rehabilitation center		R
		ERX012389	Botsomi	Tchimpounga Chimp rehabilitation center		R
		ERX012388	Agnagui	Tchimpounga Chimp rehabilitation center		R
Pan troglodytes ellioti	PRJNA189439	SRX360475	Akwaya-Jean	Wild Born	Nigerian	R
		SRX360476	Damian	Wild Born	Nigerian	R
		SRX360477	Julie_LWC21	Wild Born	Nigerian	R
		SRX360478	Koto	Wild Born	Nigerian	R
		SRX360479	Taweh	Wild Born	Nigerian	R

Continues next page

Species	Project	SRX	Sample	Origin	Observations	H558R
Pan paniscus	PRJNA189439	SRX242682				
		SRX242681	Kumbuka	Captive Born		R
		SRX241545				
		SRX241544	Salonga	Captive Born		R
		SRX241481				
		SRX241477				
		SRX241461				
		SRX241441	Natalie	Wild Born		R
		SRX241378				
		SRX241367				
		SRX241358				
		SRX241352	Bono	Wild Born		R
		SRX241312				
		SRX241311	Chipita	Wild Born		R
		SRX241308				
		SRX241307				
		SRX241305				
		SRX241304	Kombate	Wild Born		R
		SRX241303				
		SRX241302	Catherine	Wild Born		R
		SRX241295				
		SRX241294	Desmond	Wild Born		R
		SRX241293				
		SRX241292	Hermien	Wild Born		*
		SRX241291				
		SRX241290	Dzeeta	Wild Born		R
		SRX241289				
		SRX241288	Kosana	Wild Born		R
		SRX237623	Hortense	Wild Born		R
		SRX243437	LB502	Captive Born	Lymphoblastoid	R
Gorilla gorilla gorilla	PRJNA189439	SRX243504	Akiba	Wild Born		R
		SRX243505	Choomba	Wild Born		R
		SRX243506	Paki	Wild Born		*
		SRX243508	Anthal	Wild Born		R
		SRX243509	Katie_B650	Wild Born		*
		SRX243438	Kowali	Captive Born		R
		SRX243440	Bulera	Captive Born		R
		SRX243442	Azizi	Captive Born		R
		SRX243444	Suzie	Wild Born		R
		SRX243455	Banjo	Wild Born		R
		SRX243457	Mimi	Wild Born		R
		SRX243461	Delphi	Wild Born		R
		SRX243466	Kolo	Captive Born		R
		SRX243463	Coco	Wild Born		R
	PRJEB1976		ERS000016			R
	PRJNA593056	SRX7254244	Kamilah	Captive Born	Fibroblast	R/H

Continues next page

Species	Project	SRX	Sample	Origin	Observations	H558R
Gorilla gorilla diehli	PRJNA189439	SRX2343507	Nyango	Wild Born		R
Gorilla beringei graueri	PRJNA189439	SRX243529	Victoria	Captive Born	Eastern lowland gorilla	R
		SRX243528				
		SRX243532				
		SRX243531				
		SRX243530				
		SRX242688	Kaisi	Wild born	Eastern lowland gorilla	R
		SRX242687				
		SRX242686				
		SRX242685				
		SRX243453	Mkubwa	Wild born	Eastern lowland gorilla	R
Pongo pygmaeus	PRJNA189439	SRX243476	Napoleon	Wild Born	Bornean orangutan	H
		SRX243475				
		SRX243474	Tilda	Wild Born	Bornean orangutan	H/Q
		SRX243473				
		SRX243472	Sari	Captive Born	Bornean orangutan	H
		SRX243471				
		SRX243470	Temmy	Captive Born	Bornean orangutan	H
		SRX243469				
		SRX243468	Nonja	Captive Born	Bornean orangutan	H/Q
		SRX243467				
Pongo abelii		SRX243486	Buschi	Wild born	Sumatran orangutan	H
		SRX243485				
		SRX243484	Babu	Wild born	Sumatran orangutan	H
		SRX243483				
		SRX243482	Dunja	Captive Born	Sumatran orangutan	H/Q
		SRX243481				
		SRX243480	Kiki	Wild born	Sumatran orangutan	H
		SRX243479				
		SRX243478	Elsi	Wild born	Sumatran orangutan	H
		SRX243477				
Nomascus leucogenys	PRJNA232723	SRX590192	NLE-Asteriks	Captive born	female blood	R
		SRX590181	NLE-Vok	Captive born	male blood	R
	PRJNA13975	SRX119997	NLEU_NLL-600	Captive born	male blood	R
		SRX119996	NLEU_NLL-607	Captive born	female blood	R
Hylobates moloch	PRJNA82883	SRX120003	HMOL_HMO 892	Captive born	female blood	R
	PRJNA232723	SRX590190	HMO-Madena	_	male blood	R
		SRX590198	HLE-Drew	captive born	female blood	R
		SRX590196	HLE-Maung	captive born	male blood	R

Continues next page

Species	Project	SRX	Sample	Origin	Observations	H558R
Rhinopithecus roxellana	PRJNA283338	SRX1024236	RR11	Centre of Experimental Primates at Kunming Institute, China	blood adult	H
		SRX1024227	RR1		blood adult male	H
		SRX1024247	RR22		blood adult	H
		SRX1024246	RR21		blood adult	H
		SRX1024229	RR4		blood adult	H
		SRX1024237	RR12		blood adult	H
Colobus angolensis	PRJNA251421	SRX792356	CANG.PA-37697	San Diego Zoo	adult female heart	H
		SRX792360				
	SRP005434	SRX039392	Colobus monkey PR00099		Coriell DNA Male	H
Papio anubis	PRJNA433868	SRX5516791	1X1125		female	H
		SRX6809758	9045		blood	H
		SRX6809725	13951		blood	H
		SRX6809762	1X3321		blood	H
		SRX6809746	1X4080		blood	H
		SRX5516802	9841		female	H
		SRX5516820	12242		female	H
		SRX5516816	26988		female	H
Macaca mullata	PRJNA251548	SRX8532765	38158	Wisconsin National Primate Research Center	female blood	H
		SRX8532767	38160		female blood	H
		SRX8532764	38157		female blood	H
		SRX8532766	38159		female blood	H
	PRJNA382404	SRX7133280	m05200		female blood Indian breed	H
	PRJNA382404	SRX7133282	m05014		male blood Indian Breed	H
Chlorocebus sabaeus	PRJNA368714	SRX3306491	VRV1723		male blood	H
		SRX3306490	VRV1719		male blood	H
		SRX3306492	VRV1722		male blood	H
	PRJNA240242	SRX497144	VCAC-2000043-VRV0491		male acd-blood	H
		SRX497168	VCAC-2000080-VRV0501		female acd-blood	H
		SRX497142	VCAC-1998073-VRV0270		male acd-Blood	H
		SRX658822	VCAC-2005088-VRV1273		female whole blood	H
	PRJNA168527	SRX3266835	CASA-091214_gDNA_B5616_Tube1		blood	H

Continues next page

Species	**Project**	**SRX**	**Sample**	**origin**	**observations**	**H558R**
Pithecia pithecia	PRJNA399413	SRX8010141	PitPit_1_DISCOVAR	San Diego Zoo Institute for Conservation Research		H
Callithrix jacchus	PRJNA566173	SRX7047414	SAMN12783337	Wisconsin Primate Research Center	female skin cell line cj17000	H
		SRX7001670				
	PRJNA401030	SRX8843881	CJ-08-46		female fibroblast	H
Cebus capucinus imitator	PRJNA298580	SRX1560048	569822			H
		SRX1560050				
	PRJNA298580	SRX1435930	569789			H

Table S4. rs1805124 minor allele frequency distribution in all populations analysed collected from the 1KGP Phase 3. ACB- African Caribbeans in Barbados; ASW- Americans of African Ancestry in SW USA; ESN-Esan in Nigeria; GWD-Gambian in Western Divisions in the Gambia; LWK-Luhya in Webuye, Kenya; MSL-Mende in Sierra Leone; YRI-Yoruba in Ibadan, Nigeria; CLM-Colombians from Medellin, Colombia; MXL-Mexican Ancestry from Los Angeles USA; PEL-Peruvians from Lima, Peru; PUR-Puerto Ricans from Puerto Rico; CDX-Chinese Dai in Xishuangbanna, China; CHB-Han Chinese in Beijing, China; CHS-Southern Han Chinese; JPT-Japanese in Tokyo, Japan; KHV-Kinh in Ho Chi Minh City, Vietnam; CEU- Utah Residents (CEPH) with Northern and Western European Ancestry; FIN-Finnish in Finland; GBR-British in England and Scotland; IBS-Iberian Population in Spain; TSI- Tuscany in Italia; BEB-Bengali from Bangladesh; GIH-Gujarati Indian from Houston, Texas; ITU-Indian Telugu from the UK; PJL-Punjabi from Lahore, Pakistan; STU-Sri Lankan Tamil from the UK.

POPULATION	ALLELIC FREQUENCIES		GENOTYPIC FREQUENCIES		
RS1805124	T (His)	C (Arg)	TT	CC	CT
**AFRICAN**	**0.691**	**0.309**	**0.487**	**0.106**	**0.407**
**ACB**	0.719	0.281	0.542	0.104	0.354
**ASW**	0.738	0.262	0.557	0.082	0.361
**ESN**	0.783	0.217	0.596	0.030	0.374
**GWD**	0.628	0.372	0.442	0.186	0.372
**LWK**	0.697	0.303	0.485	0.091	0.424
**MSL**	0.618	0.382	0.388	0.153	0.459
**YRI**	0.671	0.329	0.426	0.083	0.491
**AMERICAN**	**0.772**	**0.228**	**0.597**	**0.052**	**0.352**
**CLM**	0.761	0.239	0.543	0.021	0.436
**MXL**	0.836	0.164	0.703	0.031	0.266
**PEL**	0.776	0.224	0.624	0.071	0.306
**PUR**	0.740	0.260	0.558	0.077	0.365
**EAST ASIAN**	**0.899**	**0.101**	**0.804**	**0.006**	**0.190**
**CDX**	0.930	0.070	0.871	0.011	0.118
**CHB**	0.874	0.126	0.748	-	0.252
**CHS**	0.905	0.095	0.819	0.010	0.171
**JPT**	0.865	0.135	0.740	0.010	0.250
**KHV**	0.924	0.076	0.848	-	0.152
**EUROPEAN**	**0.783**	**0.217**	**0.610**	**0.044**	**0.346**
**CEU**	0.818	0.182	0.657	0.020	0.323
**FIN**	0.823	0.177	0.667	0.020	0.313
**GBR**	0.775	0.225	0.593	0.044	0.363
**IBS**	0.738	0.262	0.561	**0.084**	0.355
**TSI**	0.766	0.234	0.579	0.047	0.374
**SOUTH ASIAN**	**0.727**	**0.273**	**0.532**	**0.078**	**0.391**
**BEB**	0.721	0.279	0.500	0.058	0.442
**GIH**	0.796	0.204	0.612	0.019	0.369
**ITU**	0.706	0.294	0.529	0.118	0.353
**PJL**	0.740	0.260	0.531	0.052	0.417
**STU**	0.672	0.328	0.480	0.137	0.382

Table S5. Bayesian One Sample T-Test, using the Two-sided alternative hypothesis that the superpopulation mean is not equal to the test value (H0). a- Average frequency of all populations from 1KGP, b-Average frequency of all populations within the 95% confidence interval, N- Number of populations included in the test, SD standard deviation, SE standard error of the mean, BF01 and BF10 Bayes factors of likelihood of H0 and H1, respectively. ESN-Esan in Nigeria, GWD- Gambian in Western Divisions in the Gambia, MSL-Mende in Sierra Leone, CDX-Chinese Dai in Xishuangbanna and IBS in Spain.

								95% Confidence Interval		
**Population**	**N**	**Mean** (H0)	**SD**	**SE**	**BF_{01}**	**BF_{10}**	**error %**	**Lower**	**Upper**	**Outlier sub populations**
**African**	7	0.307	0.059	0.022	2.831	0.353	6.191e-6	0.252	0.361	ESN, GWD, MSL
**American**	4	0.222	0.041	0.021	2.338	0.428	2.607e-7	0.156	0.287	
**East Asian**	5	0.100	0.029	0.013	2.515	0.398	6.831e-6	0.064	0.137	
**European**	5	0.216	0.036	0.016	2.516	0.397	7.009e-6	0.171	0.261	IBS
**South Asian**	5	0.273	0.046	0.021	2.516	0.397	7.009e-6	0.216	0.330	

Table S6. Haplotype analysis of the 1KGP data. Haplotypes were obtained through combination of variants (rs2051211, rs3922844, rs7374004, rs7374540, rs6599222, rs11710077) found to be in LD with rs1805124. Frequency and count values refer to the sum off all populations, individual population counts are shown in the respective population columns. Bold haplotypes carry the rs1805124-C allele (Arg558).

	rs2051211 rs3922844 rs7374004 rs7374540 rs1805124 rs6599222 rs11710077	Nº of risk alleles	Count	Frequency	African	American	South Asian	European	East Asian
**H1**	A_C_T_A_T_T_A	1	1132	0.226	45	187	281	304	315
**H2**	A_C_A_C_T_T_A	1	672	0.1342	117	87	88	82	298
**H3**	A_T_A_C_T_T_A	1	466	0.0931	383	42	28	6	7
**H4**	A_C_A_C_T_C_A	2	461	0.0921	161	87	89	112	12
**H5**	G_C_A_C_T_T_A	2	283	0.0565	7	17	38	49	172
**H6**	**G_T_A_A_C_T_T**	**4**	**251**	**0.0501**	**39**	**56**	**64**	**81**	**11**
**H7**	A_T_A_A_C_T_T	3	246	0.0491	58	30	80	38	40
**H8**	A_T_A_C_T_C_A	2	172	0.0343	83	28	11	49	1
**H9**	A_T_A_A_C_T_A	2	168	0.0335	75	17	16	43	17
**H10**	A_T_A_C_C_T_A	1	163	0.0325	132	13	12	6	
**H11**	G_C_T_A_T_T_A	2	137	0.0274	1	20	29	32	55
**H12**	A_C_T_A_T_T_T	2	120	0.024	3	24	31	61	1
**H13**	G_T_A_C_T_T_A	2	104	0.0208	63	10	10	4	17
**H14**	G_T_A_A_C_T_A	3	101	0.0202	12	22	23	17	27
**H15**	A_C_A_C_C_T_A	1	95	0.019	39	9	40	3	4
**H16**	G_C_A_C_T_C_A	3	81	0.0162	41	6	17	15	2
**H17**	**A_C_T_C_T_T_A**	**0**	**68**	**0.0136**	**2**	**3**	**41**	**20**	**2**
**H18**	G_T_A_C_C_T_A	2	57	0.0114	11	7	20	19	
**H19**	G_T_A_C_T_C_A	3	26	0.0052	3	7	8	8	
**H20**	**A_C_T_C_C_T_A**	**0**	**20**	**0.004**	**16**	**2**	**1**	**1**	
**H21**	A_C_A_C_T_C_T	3	19	0.0038		3	6	9	1
**H22**	G_C_T_C_T_T_A	1	15	0.003			2	4	9
**H23**	A_C_A_C_T_T_T	2	14	0.0028	1	1	11		1
**H24**	A_C_T_A_C_T_A	1	12	0.0024	6		2	1	3
**H25**	A_T_A_C_C_T_T	2	11	0.0022	11				
**H26**	G_C_A_C_C_T_A	2	11	0.0022	1	1	3	6	
**H27**	G_T_A_A_T_T_T	4	11	0.0022		4	2	5	
**H28**	G_C_T_A_T_T_T	3	10	0.002			6	4	
**H29**	A_C_A_A_T_T_A	2	9	0.0018			1	3	5
**H30**	A_C_T_A_C_T_T	2	7	0.0014			5	2	
**H31**	G_C_A_C_T_T_T	3	7	0.0014		1	2		4
**H32**	A_T_A_A_T_T_A	2	6	0.0012		1	1	2	2
**H33**	A_T_A_C_T_C_T	3	6	0.0012	1	1		3	1
**H34**	G_T_A_A_T_T_A	3	6	0.0012		2	1	3	
**H35**	G_T_A_C_T_T_T	3	6	0.0012		1	5		
**H36**	A_C_T_A_T_C_A	2	4	0.0008		1	1	2	
**H37**	A_T_A_A_T_T_T	3	4	0.0008		1		3	
**H38**	G_T_T_A_T_T_A	2	4	0.0008		1	1	2	
**H39**	A_C_A_A_C_T_T	3	3	0.0006	2	1			
**H40**	A_T_A_C_T_T_T	2	3	0.0006	1			2	
**H41**	A_T_T_A_T_T_A	1	3	0.0006		1		2	
**H42**	G_C_T_C_C_T_A	1	3	0.0006	3				

**H43**	A_T_A_C_C_C_A	2	2	0.0004	2				
**H44**	G_C_A_C_T_C_T	4	2	0.0004			1	1	
**H45**	G_T_A_C_C_C_A	3	2	0.0004	2				
**H46**	A_C_A_A_C_T_A	2	1	0.0002				1	
**H47**	A_C_T_C_T_C_A	1	1	0.0002				1	
**H48**	A_T_T_A_T_T_T	2	1	0.0002	1				
**H49**	G_C_A_A_C_T_T	4	1	0.0002			1		
**H50**	G_C_A_A_T_T_A	3	1	0.0002					1
	**Nº of haplotypes per population**				**31**	**34**	**37**	**39**	**25**
